# Influence of biceps-triceps ratio on golf swing performance

**DOI:** 10.1371/journal.pone.0307547

**Published:** 2024-07-23

**Authors:** Yue Zou, Niall MacFarlane

**Affiliations:** School of Life Sciences, University of Glasgow, Glasgow, United Kingdom; Erzurum Technical University: Erzurum Teknik Universitesi, TÜRKIYE

## Abstract

**Objective:**

This study examines how maintaining a straight leading arm affects the muscle strength balance between the biceps and triceps in golfers and its influence on golf performance.

**Methods:**

We recruited 20 male participants aged 18–45, including 10 golfers and 10 non-golfers. The participants’ average age was 25.6±6.2 years, height 1.8±0.07 m, and weight 75.6±10.2 kg. We measured isometric and isokinetic muscle strength using the Primus RS Dynamometer (BTE Technologies, Hanover, MD, USA) and assessed golf swing performance with the Optishot 2 Golf Simulator (Optishot, Brighton, MI, USA).

**Results:**

Golfers exhibited significantly greater triceps strength (P = 0.02) and a lower biceps-to-triceps strength ratio (P = 0.002) than non-golfers. Low-handicap golfers showed more centered and consistent ball impacts compared to mid-handicap golfers. There were no significant differences in swing path and face angles between low- and mid-handicap golfers. Muscle strength and the biceps-to-triceps strength ratio correlated with driving distance, as well as the frequencies of specific swing paths, face angles, and ball impact points, highlighting the complex interplay between muscle balance and swing performance.

**Conclusion:**

Greater triceps strength and a lower biceps-to-triceps strength ratio are key for maintaining a straight leading arm, especially in skilled golfers. While increased muscle strength tends to enhance driving distance, it does not necessarily improve accuracy. Consistent ball impact points may indicate higher skill levels. Future research should involve a larger, more diverse participant pool to validate these findings and further explore the complex nature of golf swing performance.

## 1 Introduction

Golf, a sport that originated in 15th-century Scotland, has evolved into a globally beloved activity, engaging an estimated 55 to 80 million enthusiasts across more than 130 countries [1]. It transcends age and socioeconomic boundaries [2]. Despite its broad player base, the sport’s fundamental goal remains unchanged: completing each hole in as few strokes as possible, with lower scores reflecting greater skill and mastery [3].

Central to golf is the mastery of the swing—a complex movement pattern that is critical to gameplay [4]. For right-handed players, the leading (left) arm plays a pivotal role in this technique [5]. It is widely endorsed that maintaining a straight leading arm throughout key phases of the swing can enhance swing speed and range of motion, thereby improving shot distance and accuracy [6]. This strategy requires keeping the leading arm straight from the backswing through the downswing to the moment of impact, ensuring consistent trajectory, power, balance, and stability [6, 7]. Crucially, this alignment optimizes shot precision and maximizes clubhead speed while avoiding excessive muscular tension, demanding significant control over the muscles around the elbow joint of the leading arm [8].

While elite golfers adeptly maintain a "single degree of freedom" in leading arm control, achieving precision and consistency, many novices struggle with maintaining a straight arm, often exhibiting bending or flexing [9]. Deviations from this control pattern, especially the transition from one to two degrees of freedom during the swing, can compromise the integrity of the swing, leading to suboptimal performance [10]. For simplicity and effectiveness in swing execution, it is recommended to maintain a straight, tension-free leading arm during critical phases of the swing. This principle is particularly advantageous for recreational players seeking to navigate the complexities of swing mechanics [10, 11].

The critical need for elbow extension in golfers presents a unique condition that contrasts with the flexor-dominant activities typical of non-golfers [12]. This unique requirement may lead to distinct characteristics in the muscle groups controlling the elbow joint. Upon conducting a thorough literature review, we found no specific studies targeting the balance of biceps and triceps strength in golfers and its impact on golf performance. While existing research has explored electromyographic activity during the golf swing and the effects of strength training on swing performance [4, 13, 14], these studies primarily focus on overall physical conditioning or the training of individual muscle groups.

Therefore, this study aims to fill the existing research gap by investigating the impact of maintaining a straight leading arm on the muscular characteristics of the biceps and triceps, which are crucial for elbow control. Additionally, by examining the biceps/triceps strength ratio and its correlation with golf performance, this research seeks to uncover the specific muscular adaptations associated with regular golf practice. Our hypothesis posits that golfers exhibit different balances of biceps and triceps strength compared with non-golfers and that this biceps/triceps strength ratio correlates with their performance.

## 2 Materials and methods

### 2.1 Participants

This study included 20 volunteer participants aged 18–45 years, comprising 10 golfers and 10 non-golfers, recruited between June 22, 2016, and July 31, 2016. Convenience sampling was used to recruit golfers from local golf clubs, while purposive sampling was employed for non-golfers to ensure a representative sample for comparative analysis. The study received approval from the MVLS College Ethics Committee (Project No: 200150167). Golfers were divided into two groups based on their handicaps: five low-handicappers (handicaps ranging from zero to seven) and five mid-handicappers (handicaps ranging from eight to 19) [15]. The handicap classification followed the World Handicap System (WHS), implemented in the UK and Scotland, which calculates a golfer’s Handicap Index by averaging the best eight scores out of the most recent 20 rounds of golf played [16]. All golfers were actively participating in recreational or competitive golf at the time of the study. Participants provided written informed consent and completed the International Physical Activity Questionnaire (IPAQ), confirming they were in good health and free from injury at the time of testing. Participant characteristics, including age, height, weight, and handedness, as well as the golf handicaps of the 10 golfers, were recorded.

### 2.2 Study design

This study utilized a cross-sectional design. Non-golfers participated in a single visit, undergoing strength measurements. Golfers, however, were involved in two visits with a minimum of 48 hours between them. During the first visit, golfers also underwent strength measurements, while the second visit focused on golf swing tests. Data collection was conducted by a trained researcher who handled test implementation and data recording.

The sample size was determined using G*Power software. Based on an estimated medium to large effect size (Cohen’s d = 0.8), commonly used in similar studies [17], we aimed for a statistical power of 0.8 and an alpha level of 0.05. According to these parameters, the calculated required sample size was approximately 26 participants per group. Due to practical constraints, a total of 20 participants were recruited for this study, which may impact the statistical power. This study was approved by the MVLS College Ethics Committee (Project No: 200150167). We confirm that informed consent was obtained from the study participants.

### 2.3 Strength measurement

The Primus RS dynamometer (BTE Technologies, Hanover, MD, USA) was used to measure the maximal strength during isometric, concentric, and eccentric contractions of the biceps and triceps muscles during the participants’ first visit. Following a warm-up, the participants were seated comfortably with their chest and thighs firmly strapped to the seat of the dynamometer to avoid compensation. The axis of rotation of the dynamometer lever arm was visually aligned with the lateral epicondyle of the humerus. Participants grasped the input handle with the forearm in pronation (for measuring the strength of the triceps brachii) or supination (for measuring the strength of the biceps brachii). Participants performed elbow flexion and extension. As all participants were right-handed, the measurements of the left arm were obtained as the left arm is the leading arm during the downswing in right-handed individuals. The participants were provided the opportunity to familiarize themselves with the equipment and exercises via submaximal contractions. To obtain the muscle strength measurements, the participants were instructed to pull toward (elbow flexion) or push away from (elbow extension) the body as hard as possible. Verbal encouragement was also provided to help participants exert their maximum effort during each contraction. The length of the dynamometer lever arm was recorded.

First, the maximal isometric strengths of the biceps and triceps were recorded. The dynamometer lever arm was locked, and the participant’s elbow was fixed at 90°. When instructed, the participants were asked to concentrically contract the biceps or triceps to produce an upward or downward force on the handle. Participants were instructed to continue their muscle contractions for five seconds and perform five maximal isometric contractions for both the biceps and the triceps with 20 seconds separating successive contractions. The order of the isometric strength measurements for the biceps and triceps for each participant was random to minimize the risk of introducing a learning bias.

Following a three-minute rest period, the participants performed isokinetic tests of the biceps and triceps at different velocities in the concentric-eccentric dynamometer mode. First, each participant performed a maximal concentric biceps or triceps contraction at 100 deg/sec followed by maximal eccentric biceps or triceps contraction at 100 deg/sec. This sequence was repeated until 10 consistent force curves were obtained. After a one-minute rest period, the isokinetic speeds were increased to 200 deg/sec and the sequence was repeated. Testing began with the elbow at 90° flexion and ended at 130° for the biceps and 10° for the triceps. Motions from 10° to 0° (0 denotes full extension) were avoided due to the potentially hazardous combination of high muscle forces and the lock-unlock mechanism of the elbow joint at full extension. Each participant performed a practice contraction at each testing speed then rested for one minute before the measurements were obtained. The order of the measurements for the biceps and triceps for each participant was random, but the order of the testing speeds was not randomized in an effort to optimize the reliability.

### 2.4 Golf swing test

On the second visit, golf swing performance was rigorously evaluated using the Optishot 2 Golf Simulator (Optishot, Brighton, Michigan, USA), equipped with a custom mat featuring sixteen 48 MHz high-speed infrared sensors for capturing detailed swing data. The setup involved ten golfers who practiced their swings by hitting plastic balls from an artificial turf tee into a net, utilizing study-provided clubs. A preliminary five-minute warm-up, allowing for ten practice shots, ensured participants’ comfort with the setup.

Swing analysis targeted five successful attempts per club: the SW (Sand Wedge), 9 Iron, 7 Iron, 5 Iron, and 3 Wood, with a randomized club sequence to eliminate bias. A swing was deemed successful if solid contact was made between the ball and the club head within the club head path. To reduce fatigue, a 30-second break was mandated between swings, with participants advised to wear comfortable athletic apparel to support optimal swing dynamics.

Participants were instructed to hit the ball as accurately and as far as possible, toward a target line, mimicking real-world golfing demands for precision and power. The Optishot 2 meticulously quantified key metrics:

- **Hitting Distance**: Estimated from club head speed and launch angle, reflecting the ball’s flight distance post-impact.
- **Swing Path**: Traced from the swing’s commencement to its follow-through, shedding light on the swing’s directional arc.
- **Face Angle**: Assessed at impact to determine the club face’s orientation relative to the target line, crucial for influencing the ball’s flight path.
- **Ball impact points**: Measured at the moment of contact between the club face and the ball, capturing the exact spot on the club face where the ball is struck.

### 2.5 Statistical analysis

Continuous data are presented as mean ± standard deviation (SD), while categorical data are presented as frequencies and percentages. The normality of continuous variables was assessed using the Shapiro-Wilk test. For normally distributed variables, Pearson’s correlation coefficient was used to examine correlations between variables. For non-normally distributed variables, Spearman’s rank correlation coefficient was applied. An analysis of variance (ANOVA) was conducted to compare means across different groups, specifically between non-golfers, golfers with low handicaps, and golfers with mid-level handicaps. Differences in swing path, face angle, and ball impact points between low- and mid-handicap golfers were compared using the chi-squared test, as these parameters are categorical. All statistical tests were two-sided, with significance set at P < 0.05. Statistical analyses were performed using SPSS version 25 (SPSS, Inc., Chicago, IL).

## 3 Results

All participants (mean age: 25.6±6.2 years, height: 1.8±0.07 m, body mass: 75.6±10.2 kg) were male and right-handed (Table 1). The average handicap in the golfer group was 13.2±5.5 for those with a mid-level handicap and 4±2.6 for those with a low handicap. All participants completed all tests in this study.

**Table 1:** Participant characteristics.

	Non-Golfers	Golfers (n = 10)	
	(n = 10)	Mid-level handicap	Low handicap
		(n = 5)	(n = 5)
Gender	All Male		
Right/left-handed		All Right-handed	
Age (years)	26 ± 5	27 ± 3	24 ± 4
Height (m)	1.8 ± 0.05	1.81 ± 0.08	1.9 ± 0.07
Weight (kg)	71.8 ± 9	75.9 ± 12.1	82.8 ± 7.95
BMI	22.6 ± 2.4	23.2 ± 3.0	23.7 ± 1.01
Handicap	NA	13.2 ± 5.5	4.0 ± 2.6

BMI: body mass index

### 3.1 Muscle Strength Comparison: Non-Golfers vs. Golfers

**Fig 1:**
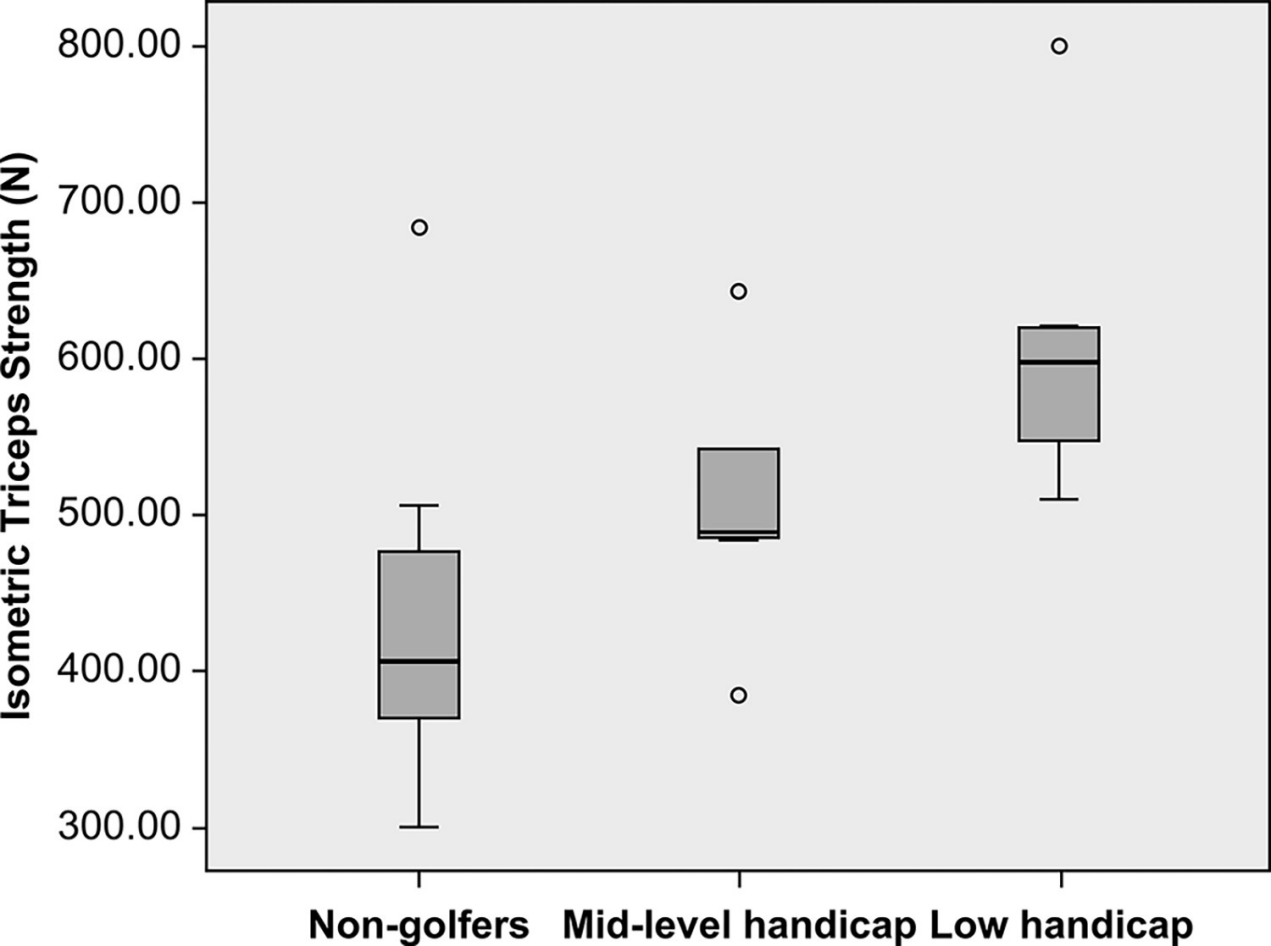
The isometric strength of non-golfers, mid-level handicap golfers and low handicap golfers.

In our study comparing muscle strength between non-golfers and golfers, significant differences were observed, particularly in isometric and isokinetic muscle strengths (Table 2, Fig 1). Golfers exhibited a notable increase in isometric triceps strength (562±112 N) compared to non-golfers (432±106 N), which was statistically significant (P = 0.02). This contrast was not evident in isometric biceps strength, where no significant difference was detected (P = 0.67).

**Table 2:** Muscle strength and muscle strength ratios of golfers and non-golfers.

		Non-golfers (n = 10)	Golfers (n = 10)	P	95%confidence interval
Isometric	Isometric biceps strength (N)	461±160	486±91	0.67	(-148, 97)
	Isometric triceps strength (N)	432±106	562±112*	0.02	(-233, -27)
	Biceps/Triceps ratio	1.05±0.12	0.87±0.11*	0.002	(0.07, 0.28)
Isokinetic 100 deg/sec	Concentric biceps strength (N)	315±136	309±93.4	0.92	(-105.8, 117.1)
	Eccentric biceps strength (N)	451±218	452±78	0.99	(161.9, 160.6)
	Concentric triceps strength (N)	346±86	401±103	0.21	(-144.4, 34.5)
	Eccentric triceps strength (N)	420±160	562±141	0.05	(-284, 0.7)
	Becc/Tcon	1.26±0.35	1.18±0.34	0.59	(-0.24, 0.41)
	Bcon/Tcon	0.90±0.26	0.78±0.23	0.28	(-0.11, 0.35)
	Becc/Tecc	1.05±0.19	0.83±0.16*	0.01	(0.07, 0.40)
Isokinetic 200 deg/sec	Concentric biceps strength (N)	254±87	247±68	0.84	(-66, 80)
	Eccentric biceps strength (N)	463±153	405±78	0.3	(-59, 176)
	Concentric triceps strength (N)	285±84	318±89	0.40	(-115, 48)
	Eccentric triceps strength (N)	407±154	589±153*	0.017	(-327, -37)
	Becc/Tcon	1.65±0.33	1.36±0.43	0.12	(-0.08, 0.65)
	Bcon/Tcon	0.90±0.16	0.79±0.18	0.16	(-0.05, 0.27)
	Becc/Tecc	1.19±0.32	0.71±0.14*	0.001	(0.24, 0.72)

*Significant difference was found between golfers and non-golfers.

Becc: eccentric biceps strength; Tcon: concentric triceps strength; Bcon: concentric biceps strength; Tecc: eccentric triceps strength

Upon analysing isokinetic strength at 100 degrees per second (deg/sec), no significant disparities were found in both concentric and eccentric biceps strength across both groups (P = 0.92 and P = 0.99, respectively). However, there was an observed trend suggesting enhanced eccentric triceps strength in golfers (562±141 N) relative to non-golfers (420±160 N), nearing statistical significance (P = 0.05). This trend continued at 200 deg/sec, with no significant difference in concentric biceps strength (P = 0.84), yet golfers demonstrated significantly greater eccentric triceps strength (589±153 N) compared to non-golfers (407±154 N), P = 0.017.

**Fig 2:**
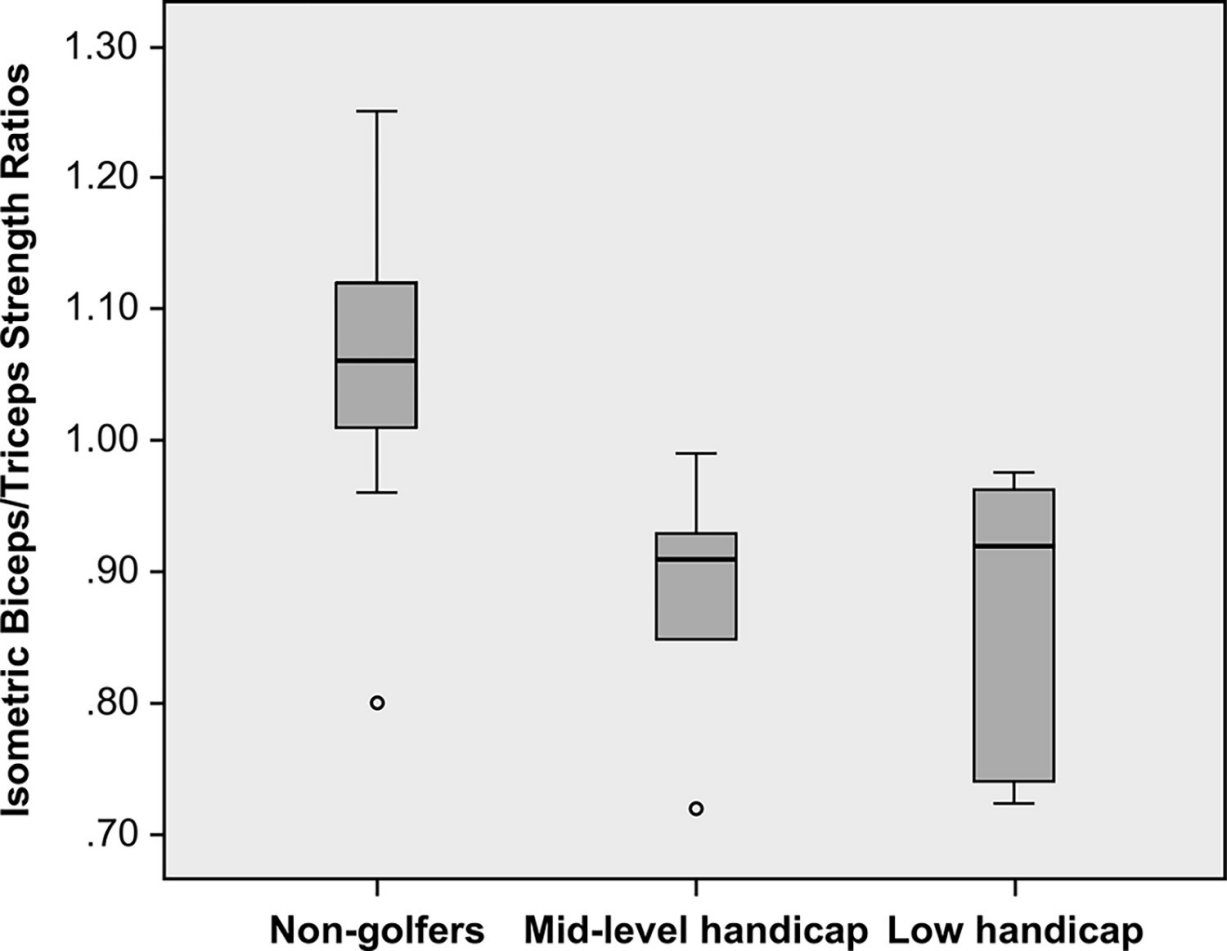
The isometric biceps/triceps strength ratios among non-golfers, mid-level handicap golfers and low handicap golfers.

Furthermore, our analysis of muscle ratios revealed a significant divergence in the biceps/triceps ratio, indicating a variance in muscle balance between the two groups (Fig 2). Golfers showed a significantly lower ratio (0.87±0.11) than non-golfers (1.05±0.12), P = 0.002. While there were no significant differences in the ratio of eccentric biceps strength to concentric triceps strength (Becc/Tcon) and the ratio of concentric biceps strength to concentric triceps strength (Bcon/Tcon) at either speed, a substantial difference was found in the ratio of eccentric biceps strength to eccentric triceps strength (Becc/Tecc) at both 100 deg/sec (0.83±0.16 vs 1.05±0.19) and 200 deg/sec (0.71±0.14 vs 1.19±0.32), with P<0.05 for both. This highlights a significant discrepancy in the balance of eccentric muscle strength, with golfers showing a reduced Becc/Tecc ratio in comparison to non-golfers.

### 3.2 Muscle Strength Comparison: Low-handicappers vs. Mid-handicappers

In our study comparing mid-handicappers to low-handicappers, we observed nuanced differences in muscle strength, with data indicating slight but not statistically significant variations (Table 3). Isometric biceps strength showed a minor increase from mid-handicappers (447.1±94.4 N) to low-handicappers (525.5±75.3 N), P = 0.19, and a similar trend was noted in triceps strength, from 508.8±94 N to 615.1±112 N, P = 0.15. The muscle balance, as reflected by the biceps/triceps ratio, was consistent across groups, 0.88±0.10 and 0.86±0.12 respectively, P = 0.83.

**Table 3:** Muscle strength and muscle strength ratios of golfers.

		Mid-handicappers (n = 5)	Low handicappers(n = 5)	P	95% confidence interval
Isometric	Isometric biceps strength (N)	447.1±94.4	525.5±75.3	0.19	(-206.2, 49.3)
	Isometric triceps strength (N)	508.8±94	615.1±112	0.15	(-260.9, 48.3)
	Biceps/Triceps ratio	0.88±0.10	0.86±0.12	0.83	(-0.15, 0.18)
Isokinetic 100 deg/sec	Concentric biceps strength (N)	301±88	317±109	0.81	(-160,128)
	Eccentric biceps strength (N)	417±80	486±65	0.17	(-176,37)
	Concentric triceps strength (N)	366±48	436±136	0.31	(-218, 78)
	Eccentric triceps strength (N)	494±85	630±160	0.13	(-323,50)
	Becc/Tcon	1.17±0.34	1.19±0.37	0.92	(-0.54, 0.49)
	Bcon/Tcon	0.84±0.29	0.73±0.16	0.48	(-0.23, 0.45)
	Becc/Tecc	0.86±0.20	0.79±0.11	0.51	(-0.17, 0.31)
Isokinetic 200 deg/sec	Concentric biceps strength (N)	242±84	251±57	0.86	(-113, 96.4)
	Eccentric biceps strength (N)	397±101	412±57	0.77	(-135,104)
	Concentric triceps strength (N)	293±117	344±48	0.39	(-182,79)
	Eccentric triceps strength (N)	526±128	653±161	0.21	(-339,85)
	Becc/Tcon	1.5±0.55	1.22±0.27	0.34	(-0.35, 0.91)
	Bcon/Tcon	0.85±0.22	0.72±0.10	0.26	(-0.12, 0.38)
	Becc/Tecc	0.76±0.12	0.65±0.14	0.20	(-0.07, 0.3)

*Significant difference was found between golfers and non-golfers.

Becc: eccentric biceps strength; Tcon: concentric triceps strength; Bcon: concentric biceps strength; Tecc: eccentric triceps strength

Further, isokinetic strength at 100 and 200 deg/sec did not reveal significant differences in concentric or eccentric strengths. The ratios of Becc/Tcon and Bcon/Tcon remained comparable across speeds and groups, indicating uniform muscle utilization. However, a closer inspection of the Becc/Tecc ratio unveiled a subtle distinction at both speeds, yet without breaching the threshold of statistical significance.

### 3.3 Golf swing performance

#### 3.3.1 Hitting distance

**Fig 3:**
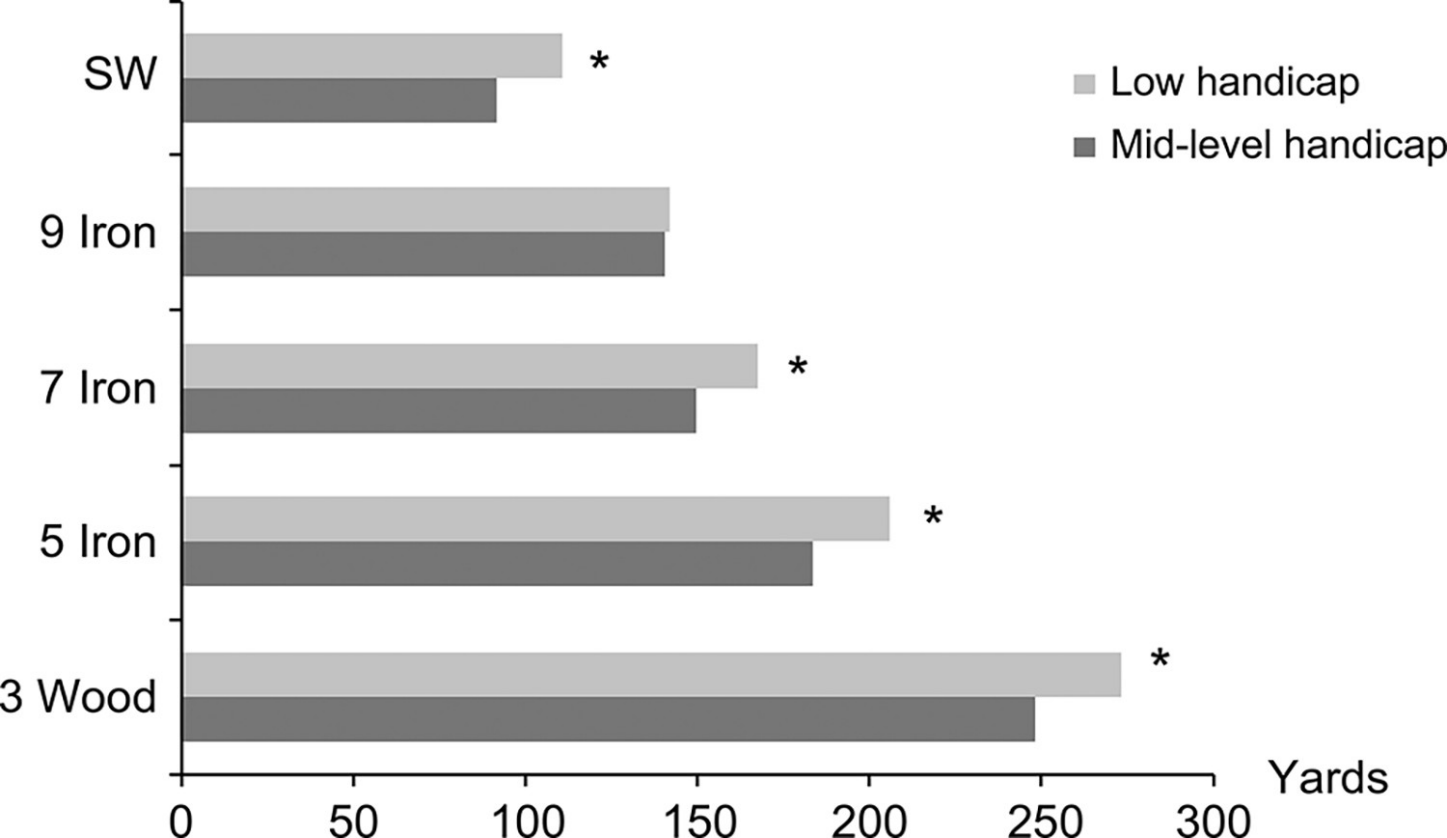
The hitting distances of 5 golf clubs for mid-level handicap golfers and low handicap golfers. * denotes a significant difference was found between them.

**Fig 4:**
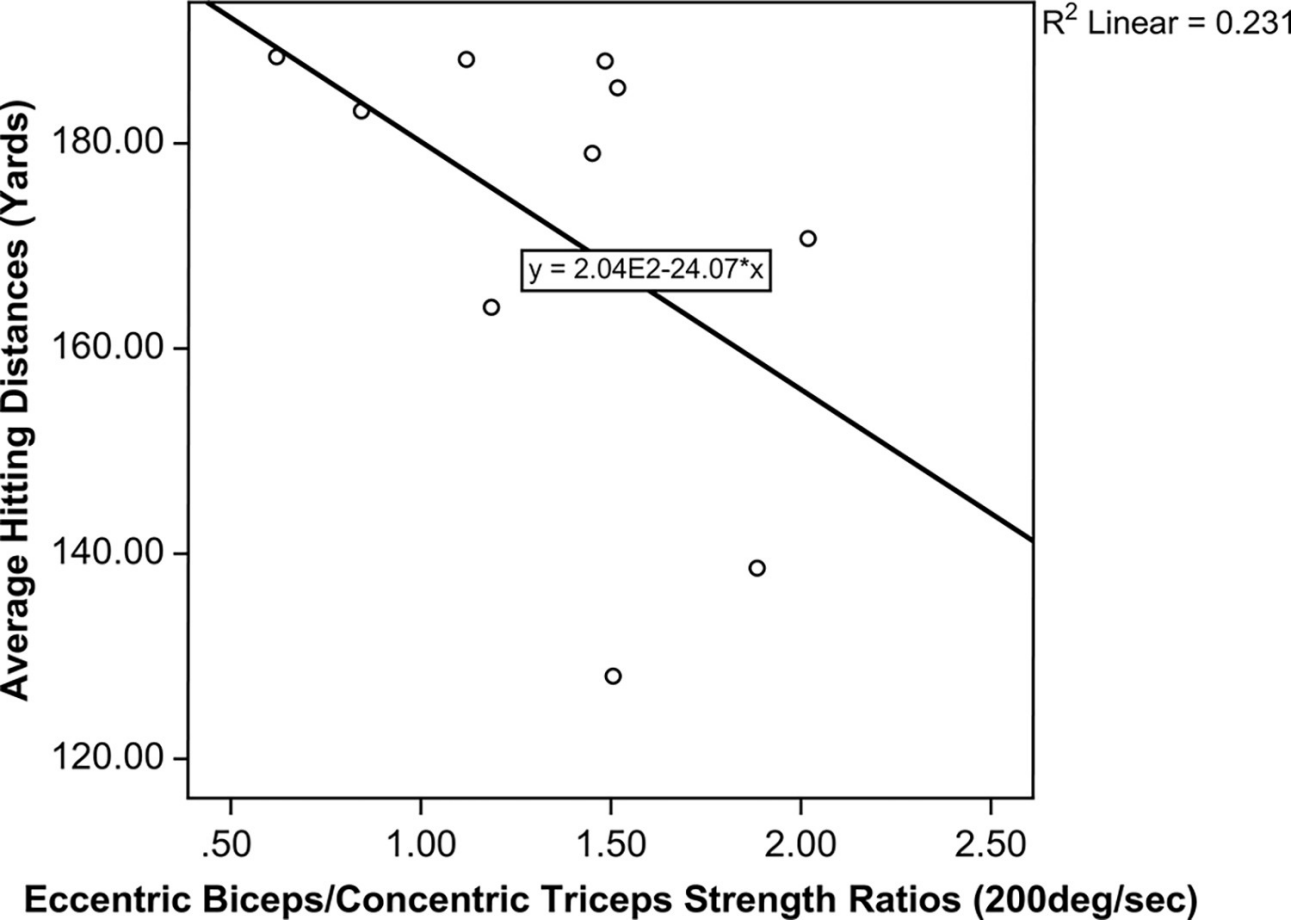
The correlation between average hitting distance and Becc/Tcon (200 deg/sec).

Except for the nine-iron, hitting distance was significantly longer among golfers with low handicaps than golfers with mid-level handicaps for each club (Fig 3). There was no difference in hitting distance between the two subgroups of golfers when the nine-iron was used (mid-handicappers: 140.7±18.2 yards, low-handicappers: 141.8±15.3 yards, P = 0.82, 95% CI: -10.65–8.49). Although triceps strength was positively correlated with hitting distance, the correlation was not statistically significant. Similarly, all strength ratios were negatively correlated with hitting distance, with the Becc/Tcon at 200 deg/sec showing the best correlation with average hitting distance, though this was also not statistically significant (Fig 4).

#### 3.3.2 Swing path

The Chi-squared test on swing path choices between low and mid-handicap golfers revealed no statistically significant differences, χ^2^ (4, N = 278) = 3.29, p = 0.51 (Table 4). Correlation analysis showed that triceps concentric strength (Tcon) at 100°/s had a significant negative correlation with the Extreme Outside/In swing path (ρ = -0.71, p = 0.02), and Becc/Tcon at 100°/s had a significant positive correlation (ρ = 0.64, p = 0.048). For the Extreme Inside/Out swing path, Tcon at 100°/s showed a marginally significant positive correlation (ρ = 0.59, p = 0.07). No significant correlations were identified for the Outside/In, Straight, and Inside/Out swing paths with any muscle strength parameters.

**Table 4:** Frequency distribution of different swing paths among mid-handicappers and low-handicappers.

Group		Frequency	Percent%
Low handicap	Extremely Outside/In	11	8.9
	Outside/In	28	22.6
	Straight	55	44.4
	Inside/Out	22	17.7
	Extremely Inside/Out	8	6.5
Mid handicap	Extremely Outside/In	7	5.6
	Outside/In	25	20.2
	Straight	56	45.2
	Inside/Out	31	25
	Extremely Inside/Out	5	4

#### 3.3.3 Face angle

Similarly, face angle distributions between low and mid-handicap golfers showed no significant differences, χ^2^ (2, N = 248) = 0.60, p = 0.74 (Table 5). Correlation analysis revealed that Tcon at 100°/s had a significant negative correlation with Close angles (ρ = -0.73, p = 0.02), and triceps eccentric strength (Tecc) at 200°/s exhibited a significant negative correlation with Square angles (ρ = -0.82, p = 0.004). Additionally, Becc/Tcon at 200°/s showed a marginally significant positive correlation with Close angles (ρ = 0.60, p = 0.07), while Tcon at 100°/s was marginally positively correlated with Open angles (ρ = 0.62, p = 0.06).

**Table 5:** Frequency distribution of different face angles among low-handicappers and mid-handicappers.

Group		Frequency	Percent%
Low handicap	Close Face	29	23.4
	Square	21	16.9
	Open Face	74	59.7
Mid handicap	Close Face	33	26.6
	Square	23	18.6
	Open Face	68	54.8

#### 3.3.4 Ball impact points

In contrast to swing path and face angle distributions, ball impact point distributions showed significant differences between low and mid-handicap golfers, χ^2^ (4, N = 248) = 10.91, p = 0.028. The expected frequencies were 1 for Heel, 9 for Slight Heel, 87 for Centre, 17.5 for Slight Toe, and 9.5 for Toe in both groups (Table 6). Correlation analysis indicated that triceps isometric strength (Tiso) had a marginally significant negative correlation with Heel impacts (ρ = -0.61, p = 0.06), while biceps isometric strength (Biso) had a significant negative correlation (ρ = -0.70, p = 0.03). Biceps concentric strength (Bcon) at 200°/s demonstrated significant negative correlations with Heel (ρ = -0.70, p = 0.03) and Slight Heel impacts (ρ = -0.71, p = 0.02), and a positive correlation with Slight Toe impacts (ρ = 0.74, p = 0.01). Tcon at 100°/s was positively correlated with Toe impacts (ρ = 0.69, p = 0.03). Tcon at 200°/s showed significant negative correlations with Heel (ρ = -0.70, p = 0.03) and Slight Heel impacts (ρ = -0.73, p = 0.02), and a positive correlation with Slight Toe impacts (ρ = 0.68, p = 0.03). Additionally, Becc/Tcon at 200°/s had a significant positive correlation with Slight Heel impacts (ρ = 0.69, p = 0.03).

**Table 6:** Frequency distribution of different ball impact points among low-handicappers and mid-handicappers.

Group		Frequency	Percent%
Low handicap	Heel	0	0
	Slightly Heel	3	2.4
	Center	93	75
	Slightly Toe	18	14.5
	Toe	10	8.1
Mid handicap	Heel	2	1.6
	Slightly Heel	15	12.1
	Center	81	65.3
	Slightly Toe	17	13.7
	Toe	9	7.3

## 4 Discussion

This study is the first to explore the impact of maintaining a straight leading arm on the balance between biceps and triceps muscles in golf swings and its subsequent influence on golf swing performance. Our findings reveal significant differences in muscular characteristics between golfers and non-golfers, particularly in triceps strength.

Golfers exhibited comparable biceps muscle strength to non-golfers but showed notably greater triceps strength, especially during eccentric contraction. This discrepancy is further highlighted by our examination of the biceps-to-triceps ratio. While the general population typically maintains a 1:1 ratio, indicating balanced strength between these muscle groups [18], golfers in our study exhibited a significantly lower ratio of approximately 0.8. This suggests that golfers possess disproportionately stronger triceps compared to their biceps, diverging from the typical strength distribution observed in non-golfers.

The observed difference in triceps strength between golfers and non-golfers implies a unique adaptation to the demands of the golf swing, particularly in maintaining a straight leading arm. This action heavily relies on triceps strength for stability and control throughout the swing motion [19]. Considering the potential implications for golf performance, targeted strength training regimens tailored to golf-specific requirements may help enhance triceps strength, especially in eccentric contractions crucial for club deceleration [20]. However, the absence of data in our study regarding the golfers’ previous strength training, particularly whether they engaged in triceps-emphasized exercises, poses a challenge in discerning whether these results stem from inherent traits of the golf game or specific training practices.

Additionally, this adaptation persists across different skill levels among golfers. When comparing low-handicap and mid-handicap golfers, notable differences in muscular strength were found. Low-handicap golfers exhibited greater strength in both biceps and triceps muscles, with a particularly pronounced advantage in triceps strength. Consequently, a lower biceps-to-triceps ratio was observed among low-handicap golfers, indicating a potential preference for stronger triceps in skilled golfers. However, the limited sample size undermines the statistical significance of these findings, highlighting the need for further research with larger cohorts. Nonetheless, these observations imply a potential association between muscular characteristics and golfing proficiency.

Confirming widely held beliefs, golfers with low handicaps typically achieve longer hitting distances, aligning with previous research findings [21, 22]. However, the unexpected finding regarding the nine-iron club raises important questions about the consistency of this relationship across various clubs. This inconsistency may suggest that factors beyond skill level alone may significantly impact hitting distance, such as club selection, variations in swing technique, or environmental conditions. These nuanced insights challenge the oversimplified assumption that hitting distance is solely determined by skill level.

Shifting focus from driving distances to swing mechanics, no significant differences in face angle and swing path were observed between low-handicap and mid-handicap golfers. However, significant differences in ball impact point distributions were noted. Low-handicap golfers exhibited more consistent and centered impacts compared to mid-handicap golfers, whose impacts were more dispersed. This aligns with previous research highlighting consistent ball impact as a key differentiator between skill levels [23–25]. These findings suggest that while technical aspects of the swing, such as face angle and swing path, are important, the consistency of ball impact is a more sensitive indicator of a golfer’s skill level.

Moreover, our study examined the relationship between muscle strength parameters and hitting distance, revealing a trend where golfers with higher triceps strength or lower biceps to triceps strength ratios tend to achieve longer driving distances. However, these correlations did not reach statistical significance, likely due to the small sample size of only 10 golfers, which reduces the statistical power and makes it difficult to detect significant differences. Future research with larger cohorts is needed to confirm these relationships and provide a more comprehensive understanding of the factors influencing hitting distance in golf.

Further correlations between muscle strength parameters and golf performance metrics were identified, demonstrating the complex interplay between muscle balance and swing precision. Higher Tcon at 100°/s was linked to reduced Extreme Outside/In swing paths, fewer closed face angles, increased toe impacts, and decreased heel impacts. Conversely, higher Becc/Tcon were associated with more frequent Extreme Outside/In swing paths and slight heel impacts, while lower ratios correlated with inside/out paths. This demonstrates the intricate relationship between muscle balance and the precision of golf swings. Nevertheless, it would be premature to conclude that muscle strength characteristics alone dictate golf swing precision. The accuracy of a golf swing is determined by a complex interplay of factors including physical conditioning, technique, equipment, and psychological influences [26]. While our data underline correlations between strength parameters and key elements of swing performance, it’s crucial to recognize the multifaceted nature of golf swing precision.

While strength parameters are crucial for understanding golf swing performance, neuromuscular control plays an equally, if not more, significant role. Studies have shown that while increased muscle strength can enhance power and driving distance, it does not automatically translate to improved swing accuracy or consistency [27–29]. A comprehensive training approach integrating both strength and neuromuscular control exercises is necessary to optimize performance. Lephart et al. (2007) demonstrated that targeted exercises focusing on neuromuscular control, such as trunk rotation and balance training, significantly improved swing mechanics and overall performance in golfers [27]. Moreover, Hetu et al. (1998) emphasized the importance of neuromuscular control in maintaining accuracy alongside increased power [28]. Fletcher and Hartwell (2004) also noted that combining strength training with exercises aimed at enhancing neuromuscular control, such as plyometrics and flexibility routines, yielded better improvements in both power and accuracy compared to strength training alone [29]. This holistic approach ensures that golfers can harness increased power without sacrificing the fine motor control needed for accurate shots.

## 5 Limitations

Several limitations in this study need to be addressed in future research. Firstly, the lack of control over the mean participant age may have influenced the findings, as muscle strength and flexibility can vary significantly across different age groups. Secondly, our study’s small sample size restricts the ability to draw definitive conclusions. Moreover, the study exclusively included young, male golfers, which limits the generalizability of the results. Future research should aim to overcome these limitations by incorporating a larger and more diverse sample.

## 6 Conclusions

This study reveals that golfers exhibit significantly greater triceps strength and a lower biceps-to-triceps strength ratio compared to non-golfers, a characteristic essential for maintaining a straight leading arm. This phenomenon is especially pronounced in skilled golfers. While increased muscle strength shows a trend towards enhancing driving distance, it does not automatically improve accuracy. Low-handicappers demonstrate more centered and consistent ball impact points compared to mid-handicappers, despite no significant differences in swing path and face angles. This consistency in ball impact points may serve as a sensitive indicator of skill levels in golfers. Overall, the findings underscore the importance of balanced muscle strength and neuromuscular control for optimizing golf performance. Future research should include a larger, more diverse participant pool to validate these findings and further explore the multifaceted nature of golf swing performance.

## References

[1] Cronin M. Sport: A very short introduction. Vol. 411. 2014: Oxford University Press, USA.

[2] Murray A. D., Daines L., Archibald D., Hawkes R. A., Schiphorst C., Kelly P., et al. The relationships between golf and health: a scoping review. British Journal of Sports Medicine, 2017. 51(1): p. 12–19. doi: 10.1136/bjsports-2016-096625 27697939 PMC5256129

[3] Davidson J.D. and Templin T.J. Determinants of success among professional golfers. Research Quarterly for Exercise and Sport, 1986. 57(1): p. 60–67.

[4] Bourgain M., Rouch P., Rouillon O., Thoreux P., & Sauret C. Golf swing biomechanics: A systematic review and methodological recommendations for kinematics. Sports, 2022. 10(6): p. 91. doi: 10.3390/sports10060091 35736831 PMC9227529

[5] Chu Y., Sell T.C., and Lephart S.M. The relationship between biomechanical variables and driving performance during the golf swing. Journal of sports sciences, 2010. 28(11): p. 1251–1259. doi: 10.1080/02640414.2010.507249 20845215

[6] Maddalozzo G.J. Sports performance series: An anatomical and biomechanical analysis of the full golf swing. Strength & Conditioning Journal, 1987. 9(4): p. 6–9.

[7] Rudy M. Golf Digest: Perfect Your Swing. 2004, Carlton Books, London.

[8] Keogh J.W. and Hume P.A. Evidence for biomechanics and motor learning research improving golf performance. Sports Biomechanics, 2012. 11(2): p. 288–309. doi: 10.1080/14763141.2012.671354 22900408

[9] Middleton, M., The performance effect of a physical guidance device on the lead-arm in golf. [A Preliminary Study]

[10] Anderson D. I., Magill R. A., Mayo A. M., & Steel K. A. Enhancing motor skill acquisition with augmented feedback, in Skill acquisition in sport. 2019, Routledge. p. 3–19.

[11] Lee T.D. and Schmidt R.A. PaR (plan-act-review) golf: Motor learning research and improving golf skills. International Journal of Golf Science, 2014. 3(1): p. 2–25.

[12] Gopura R., Kiguchi K., and Horikawa E. A study on human upper-limb muscles activities during daily upper-limb motions. International Journal of Bioelectromagnetism, 2010. 12(2): p. 54–61.

[13] Verikas A., Vaiciukynas E., Gelzinis A., Parker J., & Olsson M. C. Electromyographic patterns during golf swing: Activation sequence profiling and prediction of shot effectiveness. Sensors, 2016. 16(4): p. 592. doi: 10.3390/s16040592 27120604 PMC4851105

[14] Parker J., Lagerhem C., Hellström J., & Olsson M. C. Effects of nine weeks isokinetic training on power, golf kinematics, and driver performance in pre-elite golfers. BMC Sports Science, Medicine and Rehabilitation, 2017. 9: p. 1–12. 10.1186/s13102-017-0086-9 PMC5725976 29238597

[15] Richardson A.K. Biomechanics of the golf swing and putting stroke. 2016.

[16] Golf S. World Handicap System. 2024 [cited 2024 June 2, 2024]; Available from: https://www.scottishgolf.org/world-handicap-system.

[17] Haugen M. E., Vårvik F. T., Larsen S., Haugen A. S., van den Tillaar R., & Bjørnsen T. Effect of free-weight vs. machine-based strength training on maximal strength, hypertrophy and jump performance–a systematic review and meta-analysis. BMC Sports Science, Medicine and Rehabilitation, 2023. 15(1): p. 103. doi: 10.1186/s13102-023-00713-4 37582807 PMC10426227

[18] Kotte S. H., Viveen J., Koenraadt K. L., The B., & Eygendaal D. Normative values of isometric elbow strength in healthy adults: a systematic review. Shoulder & elbow, 2018. 10(3): p. 207–215. doi: 10.1177/1758573217748643 29796109 PMC5960876

[19] Sheehan W.B., Bower R.G., and Watsford M.L. Physical determinants of golf swing performance: A review. The Journal of Strength & Conditioning Research, 2022. 36(1): p. 289–297. doi: 10.1519/JSC.0000000000003411 31868818

[20] Alvarez M. A. R. Í. A., Sedano S., Cuadrado G., & Redondo J. C. Effects of an 18-week strength training program on low-handicap golfers’ performance. The Journal of Strength & Conditioning Research, 2012. 26(4): p. 1110–1121. doi: 10.1519/JSC.0b013e31822dfa7d 21881530

[21] Bradshaw E. J., Keogh J. W., Hume P. A., Maulder P. S., Nortje J., & Marnewick M. The effect of biological movement variability on the performance of the golf swing in high-and low-handicapped players. Research Quarterly for Exercise and Sport, 2009. 80(2): p. 185–196. doi: 10.1080/02701367.2009.10599552 19650383

[22] Keogh J. W., Marnewick M. C., Maulder P. S., Nortje J. P., Hume P. A., & Bradshaw E. J. Are anthropometric, flexibility, muscular strength, and endurance variables related to clubhead velocity in low-and high-handicap golfers? The Journal of Strength & Conditioning Research, 2009. 23(6): p. 1841–1850. doi: 10.1519/JSC.0b013e3181b73cb3 19675474

[23] Suzuki T., Sheahan J. P., Miyazawa T., Okuda I., & Ichikawa D. Comparison of TrackMan data between professional and amateur golfers at swinging to uphill and downhill fairways. The Open Sports Sciences Journal, 2021. 14(1).

[24] Bajuri M. N., Abdul Kadir M. R., Amin I. M., & Öchsner A. Biomechanical analysis of rheumatoid arthritis of the wrist joint. Proceedings of the Institution of Mechanical Engineers, Part H: Journal of Engineering in Medicine, 2012. 226(7): p. 510–520. doi: 10.1177/0954411912445846 22913098

[25] Betzler N. F., Monk S. A., Wallace E. S., & Otto S. R. Variability in clubhead presentation characteristics and ball impact location for golfers’ drives. Journal of sports sciences, 2012. 30(5): p. 439–448. doi: 10.1080/02640414.2011.653981 22272690

[26] Smith M.F. The role of physiology in the development of golf performance. Sports medicine, 2010. 40: p. 635–655. doi: 10.2165/11532920-000000000-00000 20632736

[27] Lephart S. M., Smoliga J. M., Myers J. B., Sell T. C., & Tsai Y. S. An eight-week golf-specific exercise program improves physical characteristics, swing mechanics, and golf performance in recreational golfers. The Journal of Strength & Conditioning Research, 2007. 21(3): p. 860–869. doi: 10.1519/R-20606.1 17685707

[28] Hetu F.E., Christie C.A., and Faigenbaum A.D. Effects of conditioning on physical fitness and club head speed in mature golfers. Perceptual and motor skills, 1998. 86(3): p. 811–815. doi: 10.2466/pms.1998.86.3.811 9656273

[29] Fletcher I.M. and Hartwell M. Effect of an 8-week combined weights and plyometrics training program on golf drive performance. The Journal of Strength & Conditioning Research, 2004. 18(1): p. 59–62. doi: 10.1519/1533-4287(2004)018&lt;0059:eoawcw&gt;2.0.co;2 14971982

